# Antimicrobial use trends in Canadian adult hematology/oncology inpatient wards: a multi-year exploratory analysis from 2018 to 2023

**DOI:** 10.1017/ash.2026.10359

**Published:** 2026-04-06

**Authors:** Claire Moyana, Annie-Kim Nguyen, John M. Conly, Daniel J.G. Thirion, Kevin Afra, John Bautista, Jeannette L. Comeau, Johan Delport, Yannick Émond, Gerald A. Evans, Susan Fryters, Bonita E. Lee, Xena Li, Darren Pasay, Irina Rajakumar, Mohammed A. Sarhan, Kathryn Slayter, Alena Tse-Chang, Olivia Varsaneux, Maggie Wong, Jessica J. Bartoszko

**Affiliations:** 1 Leslie Dan Faculty of Pharmacy, University of Toronto, Toronto, Canada; 2 Canadian Nosocomial Infection Surveillance Program, Public Health Agency of Canada, Ottawa, Canada; 3 Department of Medicine, Cumming School of Medicine, University of Calgary and Alberta Health Services, Calgary, Canada; 4 Faculty of Pharmacy, Université de Montréal, Montréal, Canada; 5 Department of Medicine, UBC: The University of British Columbia, Vancouver, Canada; 6 NL Health Services, Grand Falls-Windsor, Canada; 7 Department of Pediatrics, Dalhousie University, Halifax, Canada; 8 London Health Sciences Centre, London, Canada; 9 Faculty of Medicine, Université de Montréal, Montréal, Canada; 10 Division of Infectious Diseases, Department of Medicine, Queen’s University, Kingston, Canada; 11 Royal Alexandra Hospital, Alberta Health Services, Edmonton, Canada; 12 Faculty of Medicine and Dentistry, University of Alberta, Edmonton, Canada; 13 North York General Hospital, Toronto, Canada; 14 Pharmacy Services, Alberta Health Services, Vegreville, Canada; 15 Pharmacy Services, Alberta Health Services, Calgary, Canada; 16 Department of Pathology and Laboratory Medicine, UBC: The University of British Columbia, Vancouver, Canada; 17 IWK Health, Halifax, Canada; 18 Fraser Health, Surrey, Canada

## Abstract

This study explores antimicrobial use (AMU) in a voluntary sample of Canadian adult hematology/oncology wards between 2018 and 2023. Although use of third- and fourth-generation cephalosporins significantly increased, overall AMU decreased by 25%. As trends change over time, ongoing surveillance is needed to guide AMU optimization in this high-risk setting.

## Introduction

Antimicrobial use (AMU) and antimicrobial resistance (AMR) pose challenges in hematology/oncology wards. Patients with cancer are highly susceptible to infections due to immunosuppression from malignancy and chemotherapy and face infection-related mortality rates approximately three times higher than those in the general population.^1–3^ Furthermore, they may not exhibit the usual signs of infection and can deteriorate rapidly, warranting empiric broad-spectrum antibiotics in those with febrile neutropenia, which can contribute to increased AMR rates.^1,3^

This exploratory study helps address a gap in AMU and AMR surveillance data by describing AMU trends in a voluntary sample of adult hematology/oncology inpatient wards from hospitals in the Canadian Nosocomial Infection Surveillance Program (CNISP), providing novel benchmark data to inform future surveillance and antimicrobial stewardship (AMS) efforts.

## Methods

CNISP is a sentinel surveillance system and a collaborative effort between the Public Health Agency of Canada, the Association of Medical Microbiology and Infectious Disease Canada, and over 110 acute-care hospitals across 10 provinces and one territory. Hospitals that participated in AMU surveillance and submitted AMU data for adult hematology/oncology inpatient wards between 2018 and 2023 were eligible. We included hematology, oncology, bone marrow transplant, and stem cell transplant inpatient wards and compared them to medical and surgical inpatient wards combined. Herein, we refer to these as hematology/oncology and non-hematology/oncology wards, respectively.

Data on systemic antibacterial use, including Anatomical Therapeutic Chemical (ATC) codes J01, P01AB01 (metronidazole oral), and A07AA09 (vancomycin oral), were standardized to defined daily doses (DDDs) using World Health Organization (WHO) ATC/DDD values. DDDs were quantified for adult inpatient surveillance to align with WHO guidelines and allow for comparisons within Canada and internationally.^4^ AMU rates were calculated by dividing DDDs by patient days and reported as DDDs per 1,000 patient days (DDD/1000PD). Additional data related to the status and scope of prospective audit and feedback (PAF) and antibiotic preauthorization in eligible hospitals were obtained from a national cross-sectional survey.^5^

We reported on the top 10 most used antibiotics in hematology/oncology wards in 2023 and those recommended in clinical practice guidelines.^6–8^ We used the Mann-Kendall test to assess AMU rate trends in hematology/oncology wards between 2018 and 2023 and negative binomial regression to evaluate AMU rate differences between hematology/oncology and non-hematology/oncology wards. For the latter, results were reported as incidence rate ratios (IRRs) with 95% confidence intervals (CIs) adjusted for hospital-level clustering. Spearman correlation coefficients were calculated to evaluate the strength of the monotonic relationship between year and AMU rates. All analyses were conducted in R version 4.3.2 at an alpha of .05.

## Results

Ten hospitals met eligibility criteria, and those with available data each year ranged from five to nine. Hospital characteristics are reported in the Supplementary File. Briefly, hospitals with 200–500 beds were the most common until 2022 (56% to 83%). In 2023, larger hospitals (>500 beds) were the most common (67%), while teaching hospitals were predominant every year.

**Figure 1.**
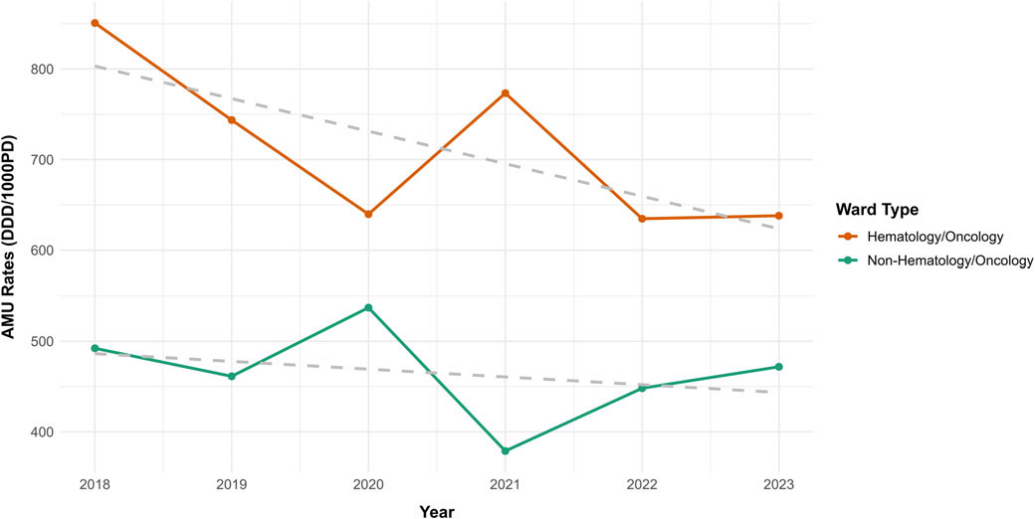
Rates of AMU (DDD/1000PD) in adult hematology/oncology and non-hematology/oncology wards (2018–2023). Hematology/oncology wards refer to hematology, oncology, bone marrow transplant, and stem cell transplant inpatient wards. Non-hematology/oncology wards refer to medical and surgical inpatient wards combined. The grey dashed line illustrates the underlying trend in antimicrobial use over time. The slope of this line corresponds to the regression coefficient, indicating the average yearly change.

Figure 1 presents overall AMU rates by hematology/oncology and non-hematology/oncology wards over time. Between 2018 and 2023, AMU declined by 25% from 850.8 to 638.0 DDD/1000PD in hematology/oncology wards (Mann-Kendall *P* = .066; r_s_ = −.77, *P* = .103) and by 4% from 492.2 to 471.8 DDD/1000PD in non-hematology/oncology wards (Mann–Kendall *P* = .354; r_s_ = −.37, *P* = .497). In 2023, the AMU rate in hematology/oncology wards was 51% (95% CI 11% to 106%) higher compared to non-hematology/oncology wards (IRR = 1.51, 95% CI: 1.11–2.06).

The Supplementary File provides overall AMU rates in adult hematology/oncology wards by antibiotic class and year. Glycopeptides demonstrated a statistically significant downward trend, while third- and fourth-generation cephalosporins demonstrated a statistically significant upward trend. Rates declined for carbapenems, quinolones, broad-spectrum penicillins, and sulfonamides/trimethoprim, although the trends were not statistically significant. Rates were relatively consistent for macrolides/lincosamides, aminoglycosides, oxazolidinones, and tetracyclines.

Table 1 reports overall AMU rates in adult hematology/oncology wards by antibiotic and year. In 2023, the 10 most frequently used antibiotics were piperacillin/tazobactam, meropenem, vancomycin, ciprofloxacin, ceftriaxone, amoxicillin/clavulanic acid, trimethoprim/sulfamethoxazole, cefazolin, azithromycin, and doxycycline. The ranking of the top five most frequently used antibiotics remained consistent over time. Between 2018 and 2023, there was a statistically significant upward trend in the use of amoxicillin/clavulanic acid and ceftriaxone. An increase was observed for cefazolin; however, there was no statistically significant trend. Rates remained relatively unchanged for doxycycline and azithromycin. While rates declined for ciprofloxacin, meropenem, piperacillin/tazobactam, and trimethoprim/sulfamethoxazole between 2018 and 2023, there was no statistically significant downward trend, unlike for vancomycin. Regarding other clinically relevant agents, a statistically significant downward trend was observed for levofloxacin; however, rates remained relatively unchanged for daptomycin, cefepime, linezolid, tigecycline, and imipenem/cilastatin. The Supplementary File provides a further breakdown by administration route.

**Table 1.** Rates of AMU (DDD/1000PD) in adult hematology/oncology wards by antibiotic and year (2018–2023)

Antibiotic	2018	2019	2020	2021	2022	2023	*P*-value^*^
Amoxicillin/clavulanic acid	35.3	38.7	17.8	39.3	40.4	44.1	**.030**
Azithromycin	22.7	18.0	13.2	24.3	21.2	22.6	1.00
Cefazolin	19.5	25.0	12.0	19.9	26.4	29.1	.066
Cefepime	7.4	6.0	7.3	11.5	8.4	11.1	.260
Ceftriaxone	29.6	23.7	37.6	37.9	38.8	46.9	**.012**
Ciprofloxacin	79.3	49.5	75.6	81.6	66.8	51.6	.354
Daptomycin	13.1	7.7	7.1	8.8	13.2	9.9	.707
Doxycycline	13.6	15.3	7.9	7.9	14.4	17.1	.566
Imipenem/cilastatin	.6	.1	.0	2.1	2.4	.7	.462
Levofloxacin	32.4	29.4	14.1	14.1	9.9	6.5	**.007**
Linezolid	3.8	3.2	1.3	2.0	2.1	3.6	1.00
Meropenem	108.9	87.1	102.4	56.8	63.8	59.5	.066
Piperacillin/tazobactam	205.4	192.0	145.7	182.2	179.0	167.3	.066
Tigecycline	1.0	.0	.0	46.0	.0	.1	.314
Trimethoprim/sulfamethoxazole	77.7	69.1	38.1	46.6	29.0	41.4	.066
Vancomycin	106.4	102.5	84.3	60.2	54.3	58.5	**.012**

* Based on Mann-Kendall trend test.

Of the 10 included hospitals, linked AMS survey data indicated that all 10 reported performing some type of PAF and six reported performing some type of antibiotic preauthorization. The majority (7/10, 70%) reported conducting PAF in hematology/oncology wards specifically, of which 86% (6/7), 100% (7/7), 86% (6/7), 53% (4/7), 71% (5/7), and 86% (6/7) reported PAF for piperacillin/tazobactam, carbapenems, fluoroquinolones, fourth-generation cephalosporins, tigecycline, and daptomycin in these wards, respectively. Half (3/6, 50%) reported performing preauthorization for meropenem, regardless of inpatient ward.

## Discussion

This exploratory analysis of a small sample of Canadian adult hematology/oncology wards provides novel data demonstrating a statistically significant upward trend in the use of third- and fourth-generation cephalosporins and a decrease in overall AMU, driven by reduced use of glycopeptides (ie, vancomycin) and, to a lesser extent, carbapenems, quinolones, broad-spectrum penicillins, and sulfonamides/trimethoprim.

In these hematology/oncology wards, lower AMU rates may be attributed to evolving AMS practices and the growing body of evidence around early antibiotic discontinuation.^9^ Meropenem use decreased by 45.4% from 2018 to 2023, potentially reflecting the impact of targeted stewardship activities (eg, carbapenem PAF), and piperacillin-tazobactam was the most used antibiotic year-over-year, consistent with its role as first-line empiric therapy for febrile neutropenia.^7^ Our findings align with those from an American academic medical center that evaluated the impact of meropenem preauthorization in hematology/oncology and transplant wards. Between 2012 and 2018, this intervention led to 42.4% reduced meropenem use and 96.2% increased piperacillin-tazobactam use, consistent with clinicians shifting toward narrower-spectrum options.^3^

This study has several limitations. Generalizability of the findings to all Canadian acute-care hospitals is uncertain since only 10 hospitals submitted AMU data from hematology/oncology wards. The ward case-mix may also limit generalizability. Only three hospitals provided data for the entire six-year study period. Changes in hospital participation year-to-year, along with pandemic-related disruptions, such as missed cancer workups and reduced hospital admissions,^10^ may have skewed temporal trend analyses and led to outliers (eg, tigecycline in 2021). Further, the study period duration may not have been sufficient to capture changes in AMU following AMS interventions, underscoring the importance of continued surveillance. Finally, aggregate ward-level data prevented further study of patient-level AMU indications, antibiotic duration and appropriateness, and clinical outcomes, including treatment failures, to assess whether reductions in AMU have unintended negative consequences.

Nonetheless, these findings may help inform AMS programs monitoring AMU in hematology/oncology wards and provide an initial benchmark for comparison. As trends differ over time and between clinical services, these findings also highlight the need for continued surveillance and targeted AMS efforts to optimize AMU in different settings. Future studies exploring how AMU trends are associated with changes in AMR within CNISP hospitals would be valuable.

## Supporting information

10.1017/ash.2026.10359.sm001 Moyana et al. supplementary material Moyana et al. supplementary material

## Supplementary material

The supplementary material for this article can be found at https://doi.org/10.1017/ash.2026.10359.
