# Supplementary material for: Antimicrobial use trends in Canadian adult hematology/oncology inpatient wards: a multi-year exploratory analysis from 2018 to 2023
Source: Antimicrob Steward Healthc Epidemiol. 2026 Apr 6;6(1):e90. doi: 10.1017/ash.2026.10359 (PMC13104512; doi:10.1017/ash.2026.10359)
Supplement: Moyana et al. supplementary material [file S2732494X26103593sup001.docx]

Contents

[Distribution of hospital characteristics from 2018–2023. 2](#_Toc218608794)

[Antimicrobial Use Rates (DDDs per 1,000 patient-days) in Adult Hematology/Oncology Wards by Antibiotic Class and Year (2018–2023). 3](#_Toc218608795)

[Antimicrobial Use Rates (DDDs per 1,000 patient-days) in Adult Hematology/Oncology Wards by Antibiotic, Administration Route and Year (2018–2023). 4](#_Toc218608796)

Distribution of hospital characteristics from 2018–2023.

| **Characteristic** | **Category** | **2018**  **N=6^1^** | **2019**  **N=5^1^** | **2020**  **N=6^1^** | **2021**  **N=9^1^** | **2022**  **N=7^1^** | **2023**  **N=6^1^** |
| --- | --- | --- | --- | --- | --- | --- | --- |
| **Number of Beds** | <200 | 1 (17%) | 0 (0%) | 0 (0%) | 0 (0%) | 0 (0%) | 1 (17%) |
|  | 200-500 | 4 (67%) | 4 (80%) | 5 (83%) | 5 (56%) | 4 (57%) | 1 (17%) |
|  | >500 | 1 (17%) | 1 (20%) | 1 (17%) | 4 (44%) | 3 (43%) | 4 (67%) |
| **Teaching Status** | Yes | 5 (83%) | 4 (80%) | 5 (83%) | 7 (78%) | 6 (86%) | 5 (83%) |
|  | No | 1 (17%) | 1 (20%) | 1 (17%) | 2 (22%) | 1 (14%) | 1 (17%) |
| **Region** | Central ^2^ | 1 (17%) | 1 (20%) | 2 (33%) | 3 (33%) | 3 (43%) | 2 (33%) |
|  | Eastern ^3^ | 2 (33%) | 2 (40%) | 2 (33%) | 2 (22%) | 2 (29%) | 2 (33%) |
|  | Western^4^ | 3 (50%) | 2 (40%) | 2 (33%) | 4 (44%) | 2 (29%) | 2 (33%) |

^1^ N = number of hospitals included that year

For the purposes of this analysis, Canadian provinces were grouped as follows:

^2^ Central Canada: Ontario, Quebec

^3^ Eastern Canada: Nova Scotia, Newfoundland and Labrador, Prince Edward Island, New Brunswick

^4^ Western Canada: British Columbia, Alberta, Saskatchewan, Manitoba

Antimicrobial Use Rates (DDDs per 1,000 patient-days) in Adult Hematology/Oncology Wards by Antibiotic Class and Year (2018–2023).

| Antibiotic Class | 2018 | 2019 | 2020 | 2021 | 2022 | 2023 | p-value* |
| --- | --- | --- | --- | --- | --- | --- | --- |
| Aminoglycosides | 2.4 | 1.3 | 1.2 | 4.5 | 3.2 | 2.7 | 0.707 |
| Broad-spectrum penicillins† | 252.7 | 242.7 | 179.9 | 233.9 | 227.7 | 220.1 | 0.066 |
| Carbapenems | 113.6 | 90.6 | 105.6 | 63.5 | 70.0 | 64.1 | 0.066 |
| Cephalosporins (3rd gen.) | 39.2 | 30.0 | 45.2 | 45.0 | 46.2 | 52.2 | **0.030** |
| Cephalosporins (4th gen.) | 16.6 | 19.0 | 25.1 | 35.1 | 28.4 | 60.8 | **0.012** |
| Glycopeptides | 106.4 | 102.5 | 84.3 | 60.2 | 54.3 | 58.7 | **0.012** |
| Macrolides/lincosamides | 28.1 | 20.4 | 19.7 | 28.0 | 26.0 | 25.9 | 0.707 |
| Oxazolidinones | 5.4 | 3.9 | 1.4 | 3.8 | 2.7 | 6.8 | 1.00 |
| Quinolones | 128.5 | 88.2 | 96.0 | 102.7 | 83.5 | 65.4 | 0.066 |
| Sulfonamides/trimethoprim | 77.7 | 69.1 | 38.1 | 46.6 | 29.0 | 41.4 | 0.066 |
| Tetracyclines | 16.5 | 17.4 | 14.4 | 101.3 | 15.8 | 21.5 | 0.707 |

*Based on Mann-Kendall trend test

†Combinations of penicillins, including beta-lactamase inhibitors

Antimicrobial Use Rates (DDDs per 1,000 patient-days) in Adult Hematology/Oncology Wards by Antibiotic, Administration Route and Year (2018–2023).

| Antibiotic | Route | 2018 | 2019 | 2020 | 2021 | 2022 | 2023 | p-value* |
| --- | --- | --- | --- | --- | --- | --- | --- | --- |
| Amoxicillin/clavulanic acid | Oral | 26.9 | 30.7 | 15.5 | 43.3 | 46.4 | 50.7 | - |
|  | Parenteral | 0.0 | 0.0 | 0.0 | 0.1 | 0.0 | 1.8 | - |
|  | SDNS | 36.2 | 27.6 | 13.9 | 0.0 | 0.0 | 0.0 | - |
|  | Overall | 35.3 | 38.7 | 17.8 | 39.3 | 40.4 | 44.1 | **0.030** |
| Azithromycin | Oral | 15.9 | 12.5 | 10.3 | 19.6 | 17.8 | 19.5 | - |
|  | Parenteral | 2.2 | 2.9 | 2.0 | 6.1 | 4.9 | 4.4 | - |
|  | SDNS | 19.8 | 9.4 | 8.1 | 4.8 | 5.9 | 12.3 | - |
|  | Overall | 22.7 | 18.0 | 13.2 | 24.3 | 21.2 | 22.6 | 1.00 |
| Cefazolin | Parenteral | 19.5 | 25.0 | 12.0 | 19.9 | 26.4 | 29.1 | 0.066 |
| Cefepime | Parenteral | 7.4 | 6.0 | 7.3 | 11.5 | 8.4 | 11.1 | 0.260 |
| Ceftriaxone | Parenteral | 29.6 | 23.7 | 37.6 | 37.9 | 38.8 | 46.9 | **0.012** |
| Ciprofloxacin | Oral | 55.8 | 32.6 | 44.1 | 76.9 | 61.9 | 48.8 | - |
|  | Parenteral | 12.3 | 6.2 | 5.9 | 9.5 | 9.5 | 5.9 | - |
|  | SDNS | 48.0 | 37.3 | 73.4 | 24.7 | 26.0 | 30.6 | - |
|  | Overall | 79.3 | 49.5 | 75.6 | 81.6 | 66.8 | 51.6 | 0.354 |
| Daptomycin | Parenteral | 13.1 | 7.7 | 7.1 | 8.8 | 13.2 | 9.9 | 0.707 |
| Doxycycline | Oral | 7.0 | 5.5 | 5.2 | 8.0 | 13.2 | 16.8 | - |
|  | Parenteral | 0.0 | 0.0 | 0.0 | 0.1 | 0.1 | 0.0 | - |
|  | SDNS | 28.4 | 33.9 | 8.1 | 5.7 | 22.2 | 18.8 | - |
|  | Overall | 13.6 | 15.3 | 7.9 | 7.9 | 14.4 | 17.1 | 0.566 |
| Imipenem/cilastatin | Parenteral | 0.6 | 0.1 | 0.0 | 2.1 | 2.4 | 0.7 | 0.462 |
| Levofloxacin | Oral | 21.5 | 18.7 | 10.4 | 11.8 | 8.6 | 5.0 | - |
|  | Parenteral | 3.5 | 1.5 | 1.1 | 2.0 | 2.1 | 1.4 | - |
|  | SDNS | 32.2 | 31.8 | 11.3 | 15.3 | 2.0 | 5.7 | - |
|  | Overall | 32.4 | 29.4 | 14.1 | 14.1 | 9.9 | 6.5 | **0.007** |
| Linezolid | Oral | 0.9 | 2.0 | 0.8 | 1.6 | 1.2 | 1.4 | - |
|  | Parenteral | 2.4 | 0.8 | 0.3 | 0.6 | 1.0 | 2.0 | - |
|  | SDNS | 2.1 | 1.3 | 0.8 | 0.4 | 0.8 | 2.4 | - |
|  | Overall | 3.8 | 3.2 | 1.3 | 2.0 | 2.1 | 3.6 | 1.00 |
| Meropenem | Parenteral | 108.9 | 87.1 | 102.4 | 56.8 | 63.8 | 59.5 | 0.066 |
| Piperacillin/tazobactam | Parenteral | 205.4 | 192.0 | 145.7 | 182.2 | 179.0 | 167.3 | 0.066 |
| Tigecycline | Parenteral | 1.0 | 0.0 | 0.0 | 46.0 | 0.0 | 0.1 | 0.314 |
| Trimethoprim/sulfamethoxazole | Oral | 54.0 | 49.8 | 29.1 | 44.0 | 29.7 | 45.9 | - |
|  | Parenteral | 8.2 | 7.5 | 1.9 | 6.7 | 3.1 | 3.1 | - |
|  | SDNS | 66.5 | 41.0 | 31.0 | 0.0 | 0.0 | 0.0 | - |
|  | Overall | 77.7 | 69.1 | 38.1 | 46.6 | 29.0 | 41.4 | 0.066 |
| Vancomycin | Oral | 15.4 | 18.5 | 7.8 | 4.7 | 3.8 | 3.9 | - |
|  | Parenteral | 91.0 | 84.0 | 58.4 | 53.1 | 45.5 | 46.2 | - |
|  | SDNS | 0.0 | 0.0 | 37.2 | 31.1 | 42.9 | 54.4 | - |
|  | Overall | 106.4 | 102.5 | 84.3 | 60.2 | 54.3 | 58.5 | **0.012** |

*Based on Mann-Kendall trend test

Note: SDNS indicates that the site did not specify the antimicrobial route of administration. For antimicrobials where only a single route appears in the table, this reflects that the antimicrobial is exclusively administered through that route. An absence of a rate for a given antimicrobial–route combination (recorded as 0.0) indicates that the site either did not collect or report data for that route of administration, or that the route was not used during the study period.
